# Phylogenetic diversity and conservation of crop wild relatives in Colombia

**DOI:** 10.1111/eva.13295

**Published:** 2021-09-16

**Authors:** Carlos E. González‐Orozco, Chrystian C. Sosa, Andrew H. Thornhill, Shawn W. Laffan

**Affiliations:** ^1^ Corporación Colombiana de Investigación Agropecuaria (AGROSAVIA) Centro de Investigación La Libertad Villavicencio Colombia; ^2^ Departamento de Ciencias naturales y Matemáticas Pontificia Universidad Javeriana Cali Cali Colombia; ^3^ Grupo de Investigación en Evolución Ecología y Conservación EECO Programa de Biología Facultad de Ciencias Básicas y Tecnologías Universidad del Quindío Armenia Colombia; ^4^ Environment Institute The University of Adelaide Adelaide SA Australia; ^5^ Department for Environment and Water State Herbarium of South Australia Botanic Gardens and State Herbarium Adelaide SA Australia; ^6^ Earth and Sustainability Science Research Centre School of Biological, Earth and Environmental Sciences The University of New South Wales Kensington NSW Australia

**Keywords:** agriculture, crop wild relatives, evolution, neotropics, phylogenetic diversity, species richness

## Abstract

Crop wild relatives (CWR) are an important agricultural resource as they contain genetic traits not found in cultivated species due to localized adaptation to unique environmental and climatic conditions. Phylogenetic diversity (PD) measures the evolutionary relationship of species using the tree of life. Our knowledge of CWR PD in neotropical regions is in its infancy. We analysed the distribution of CWR PD across Colombia and assessed its conservation status. The areas with the largest concentration of PD were identified as being in the northern part of the central and western Andean mountain ranges and the Pacific region. These centres of high PD were comprised of predominantly short and closely related branches, mostly of species of wild tomatoes and black peppers. In contrast, the CWR PD in the lowland ecosystems of the Amazon and Orinoquia regions had deeply diverging clades predominantly represented by long and distantly related branches (i.e. tuberous roots, grains and cacao). We categorized 50 (52.6%) of the CWR species as ‘high priority’, 36 as ‘medium priority’ and nine as ‘low priority’ for further ex‐situ and in situ conservation actions. New areas of high PD and richness with large ex‐situ gap collections were identified mainly in the northern part of the Andes of Colombia. We found that 56% of the grid cells with the highest PD values were unprotected. These baseline data could be used to create a comprehensive national strategy of CWR conservation in Colombia.

## 1 INTRODUCTION

There are an estimated 50,000 species of crop wild relatives (CWR) worldwide, of which 800 are considered as the highest priority for conservation (Maxted et al., 2012). However, the number of CWR species with importance for conservation in the tropics is unclear. Colombia, a neotropical subregion, is an important geographic area for the origins of Andean and tropical South American crops (Khoury et al., 2016; Lombardo et al., 2020; Vélez et al., 2016). According to recent studies focusing on global conservation of CWR (Castañeda‐Álvarez et al., 2016; Vincent et al., 2019), Colombia is not considered a high priority region for sourcing CWR of global importance. In terms of CWR global hotspots, Colombia does not appear in the areas of high CWR diversity and centres of crop origins (Kell et al., 2017; Khoury, Carver, et al., 2019; Maxted et al., 2007; Maxted & Vincent, 2021). Consequently, Colombia remains largely unexplored as a region of importance for the biodiversity of CWR that are not yet formally recognized but which possess great potential. This might be because Colombia's CWR are poorly documented and do not represent a large proportion of the main global food crops or their centres of origin (Cañas‐Gutiérrez et al., 2019; Couvreur et al., 2007; González‐Orozco et al., 2020; Jarvis et al., 2008; Ocampo et al., 2010).

Biodiversity is often measured by counting the number of species in an area (species richness). This is an informative metric but does not indicate the diversity of the tree of life in an area (Mishler, 2010). A key method to quantify evolutionary diversity in the tree of life is phylogenetic diversity (PD; Faith, 1992). PD measures evolutionary diversity by summing the lengths of branches connecting the tips of a phylogenetic tree, normally to the root of the tree but sometimes only to the most recent common ancestor. The PD metric is a key tool in the identification of evolutionary relationships across space, hence improving our capacity to measure important genetic resources (Faith, 2013). PD is recognized as one of the flagship conservation metrics to maximize protection of biodiversity (Daru et al., 2019; Forest et al., 2007; Gumbs et al., 2020; Laity et al., 2015; Rosauer et al., 2017; Tolley et al., 2016; Zhang et al., 2016). The Convention on Biological Diversity (CBD) declared the tree of life, or PD, as an effective way of preserving biodiversity for people because it maintains the benefits of nature to humanity (Gumbs et al., 2020). In practical terms, PD not only represents a variety of evolutionary features and heritage of species but also safeguards key sections of the tree of life and therefore the potential uses of unexplored biodiversity under a current changing environment and its future threats.

Although PD is recognized as a key biodiversity indicator, finding available data to quantify PD in tropical regions is a challenge. This aspect, combined with the fact that Colombia is a potential source of unexplored CWR diversity, makes our case study a research priority for the in situ conservation of native genetic resources. To illustrate the importance of documenting CWR diversity in regions such as Colombia, here we will mention the example of the cacao biological expeditions, CacaoBIO (https://bit.ly/3j72GKz). The CacaoBIO project was founded by the Colombian government and explored patterns of diversity and distribution of cacao CWR in the Amazon and Choco biogeographic regions of Colombia, identifying 22 of the 26 reported taxa of wild cacao in the world by revisiting Colombian regions that have not been explored for more than 50 years due to armed conflict. A key result of this study was finding a large diversity of wild *Theobroma cacao* L. as well as extant plants of the genus *Herrania* which is sister to *Theobroma* (González‐Orozco, Sanchez, et al., 2020). The PD of wild cacao in Colombia has not yet been fully quantified but sampling a wide variety of CWR gene pools increases the chances of using this cacao CWR biodiversity in future genetic studies.

There are many PD‐based approaches that can be applied, depending on the extent and target group under study. However, there is not a single most comprehensive PD metric (Cadotte et al., 2010). When applied spatially, these alternate metrics fall under the umbrella of spatial phylogenetics (Azevedo et al., 2020; Gonzalez‐Orozco et al., 2016; Laffan, 2018; Laffan et al., 2010; Mishler et al., 2020; Scherson et al., 2017; Thornhill et al., 2017). In this research, we apply some of the derived measures of spatial PD developed and tested extensively in different biological groups, continents, and regions (Mienna et al., 2020; Mishler et al., 2020). Relative phylogenetic diversity (RPD) identifies locations with unusual concentrations of long or short branches of the tree of life by comparing PD calculated using the observed tree with that calculated using a tree with the same topology but where all branch lengths are set to the mean non‐zero branch length. This type of information helps to reveal evolutionary patterns such as where assemblages are more closely or distantly related over time (Thornhill et al., 2016). A key part of spatial phylogenetics is the use of randomization approaches to assess significance of observed diversity patterns, enabling the identification of distributions that are more extreme than expected under a random scenario (Mishler et al., 2014).

Regarding the application of PD to close relatives of crops, Jovovic (2020) noted that CWR are a fundamental element in modern agriculture because they provide important genes for plant breeders. Therefore, it is important to investigate the use of PD for the conservation of wild relatives. Particularly, a stable conservation status of the CWR species in Colombia is lacking. One of the newly developed tools to assess species status in conservation is the GapAnalysis R package (Carver et al., 2021). Gap analysis assessments are extremely useful for conservation planning of genetic resources because they help identify areas where more species collections are required (Khoury, Carver, et al., 2019). However, they have not yet been used to explore the conservation status of PD.

In this study, we identify the major centres of PD CWR in Colombia. We also investigate how well‐conserved high areas of PD and species richness are, as well as identify regions where additional areas for further collecting are needed to plan ex‐situ and in situ conservation of Colombia's CWR.

## 2 MATERIALS AND METHODS

### 2.1 Study region

The study region is continental Colombia, excluding islands and archipelagos (Figure 2a). Colombia is composed of two dominions (Pacific and Boreal Brazilian) and six biogeographic provinces (Chocó‐Darién, Guajira, Magdalena, Páramo, Sabána, and Imerí) (Figure S1c; González‐Orozco, 2021).

### 2.2 Species occurrences

To determine which CWR species to include in our study, we used the species list and distribution records of CWR for Colombia from the Global Biodiversity Information Facility (GBIF) database version 1.12, generated by the International Centre for Tropical Agriculture (CIAT)‐(CWRODC, 2018). To create the spatial data set, we extracted all geocoded CWR species records for Colombia from the CWR GBIF database (GBIF, 2019). We used the accepted CWR taxonomic names listed in CWR GBIF to filter the records to be extracted from version 4 of the Botanical Information and Ecology Network (BIEN) data set (Maitner et al., 2017) and generated a single data set that comprised all CWR records from both sources. The raw data set contained a total of 357,582 records comprising 241 CWR species (GBIF: 17,666 records, 227 species; BIEN: 339,916 records, 207 species), 49 plant genera and 27 families. From these, 185 species of CWR were used in the PD analysis once naturalized species were excluded. The raw data set was filtered to remove occurrences without geographic coordinates, records falling outside continental Colombia and non‐native and agricultural crops. The corrected and cleaned data set contained a total of 10,376 records comprising 185 CWR species native to Colombia (see Table S2 for genus list; and Dataset S1 for final spatial data and species list with botanical families). These data were imported into Biodiverse version 3.0 (Laffan et al., 2010) and aggregated to square grid cells with a spatial resolution of 0.1° (~10 km). This resulted in a total of 1801 grid cells spanning continental Colombia.

### 2.3 Assembly of molecular data and phylogenetic analyses

A list of Colombian CWR plants was used to search GenBank using Matrix Maker (Freyman & Thornhill, 2016). Sequences of seven loci were searched for—*trn*L, *mat*K, ITS, *rbc*L *trnL*‐*trn*F, *atp*B and *mat*R (accessions for each locus are in Table S3). Individual alignments of each locus were created using MaffT version 7 (Katoh, 2013) and concatenated using SequenceMatrix (Vaidya et al., 2011). A maximum‐likelihood analysis was performed on the concatenated alignment using RAxML in the Cipres Portal (Miller et al., 2009). Most of the nodes of the phylogeny have bootstrap support values of 100% across the tree (Figure S2). The phylogenetic tree is available in Dataset S2. The resulting tree was exported and converted to nexus format using FigTree version 1.3 (Rambaut, 2009).

### 2.4 Species richness and sampling redundancy analyses

We used the Biodiverse software (Laffan et al., 2010), version 3.0, to calculate redundancy and species richness (SR) indices for each 0.1‐degree grid cell. Redundancy represents the ratio of species to samples per grid cell and has values in the interval [0,1]. Values close to 1 are well sampled, while those near zero have poor sample redundancy. Species richness represents the total number of unique taxa in each grid cell.

### 2.5 Phylogenetic diversity analyses

Biodiverse version 3.0 (Laffan et al., 2010) was used to calculate a set of spatial phylogenetic indices for the 0.1‐degree grid cells for the combined GBIF and BIEN data set. As defined in the introduction, observed PD and RPD were calculated using branches connecting the terminals to the root branch. The statistical significance of the PD and RPD values was estimated using a randomization process in which taxa were randomly allocated across the landscape, but where the range of each taxon and the richness of each cell were held constant (Laffan & Crisp, 2003; Mishler et al., 2014; Thornhill et al., 2016). A total of 999 random realizations were run and compared against the observed values to estimate rank‐relative significance of observed PD (one‐tailed high test) and RPD (two‐tailed test). A significantly high PD score indicates there is more of the tree in a region than expected, while a significantly low PD score indicates there is less of the tree in a region than expected. For RPD, a significantly high value for a region indicates an over‐representation of long branches, while a significantly low value indicates an over‐representation of short branches (Mishler et al., 2014).

### 2.6 Species distribution modelling

In preparation for the gap analysis, the maximum entropy (MaxEnt) algorithm (Phillips et al., 2006) was applied to produce potential ecogeographic suitability models for 101 of the 185 species of CWR included in our study (Table S1). Thirty per cent of the occurrences per species were used to conduct a random test of the samples. The default setting was used for the remainder of the modelling parameters. The 89 species for which the MaxEnt models were not run had insufficient conditions to satisfy the required parameters. The AUC value used to optimize predictability was >0.7, and replicates were set to 5. Seven climate variables representing the average climatic history of Colombia from 1980 to 2010 (mean annual precipitation, average temperature, minimum and maximum temperature, relative humidity, solar radiation, wind speed) with a spatial resolution of 3 × 3 km were used as predictors for the MaxEnt modelling (Agrosavia, 2014; Alzate‐Velásquez, 2017, 2018; González‐Orozco, Porcel, et al., 2020). Suitability values >= 0.5 were labelled as 1 and those <0.5 were labelled as 0 to generate a binary classification required as an input of the GapAnalysis. Further, using the nearest neighbour resampling method, the MaxEnt rasters were masked using a Colombia protected areas layer using a buffer of 5km following the default suggested by Khoury, Amariles, et al. (2019).

### 2.7 Conservation gap analysis of CWR species

We assessed the degree of representativeness of 101 species of CWR in an ex‐situ conservation system using the R package GapAnalysis (Carver et al., 2021). The 89 species of CWR that did not fulfil the gap analyses criteria were assigned as high priority for further collecting in both in situ and ex‐situ systems. This way we were able to use the full set of 185 species used in the PD and species richness analyses. This approach estimates eight metrics of conservation representativeness. For both the ex‐situ and in situ metrics, the indices calculated were sampling score (SRSex‐in), geographic score (GRex‐in), ecological score (ERSex‐in), and the final conservation score (FCSex‐in). A further index is a combined metric, or FSC‐mean, which was calculated for 95 species by averaging the final ex‐situ FCSex and in situ FCSin scores. To assess the ex‐situ representativeness of the species used in the analysis, the online plant genetic resource platform ‘Genesys’ (https://www.genesys‐pgr.org/) was searched for the records included in our sample that was conserved in any of the germplasm collections mentioned in Genesys. The FAO WIEWS data set was also assessed as part of the review for building our data set of germplasm collections, but its information was already contained in Genesys. The final combined per‐species scores were assigned a series of status conservation categories according to Khoury, Amariles, et al. (2019). Finally, a predicted species richness gap analysis was calculated summing up the SDM binary MaxEnt rasters for the 101 CWR species.

For the cases where CWR species had ex‐situ gap analysis results, a PD conservation gap richness indicator was developed. The observed PD raster (10 km) was resampled to fit the spatial resolution of the modelled species (5 km) using a bilinear function. Then, the PD and gap richness maps were standardized dividing by their respective maximum values to obtain values from 0 to 1. Finally, the standardized PD and ex‐situ gap richness for further collecting were averaged to generate the PD conservation gap richness indicator. This new spatial indicator of PD conservation gap richness allowed us to prioritize areas where there are important ex‐situ sites that require further ex‐situ collecting (i.e. germplasm banks). To complement the PD gap richness indicator, a PD and predicted species richness indicator was calculated as the sum of the binary SDMs for the 101 species instead of using gap richness. This PD and predicted species richness indicator allowed us to prioritize areas where there is a greater concentration of both species richness and PD.

### 2.8 Conservation assessment of PD

An assessment of PD conservation was conducted using the spatial intersect tool in QGIS. Grid cells with the highest values of observed SR and PD (top 5–95 quantile), and significantly high and low grid cells of PD and RPD that overlapped with the protected areas of each department in Colombia was counted. Consequently, a percentage of regional representativeness of PD inside protected areas per department was estimated. This assessment did not include any information from the Gap Analysis.

## 3 RESULTS

### 3.1 Phylogenetic tree

**FIGURE 1:**
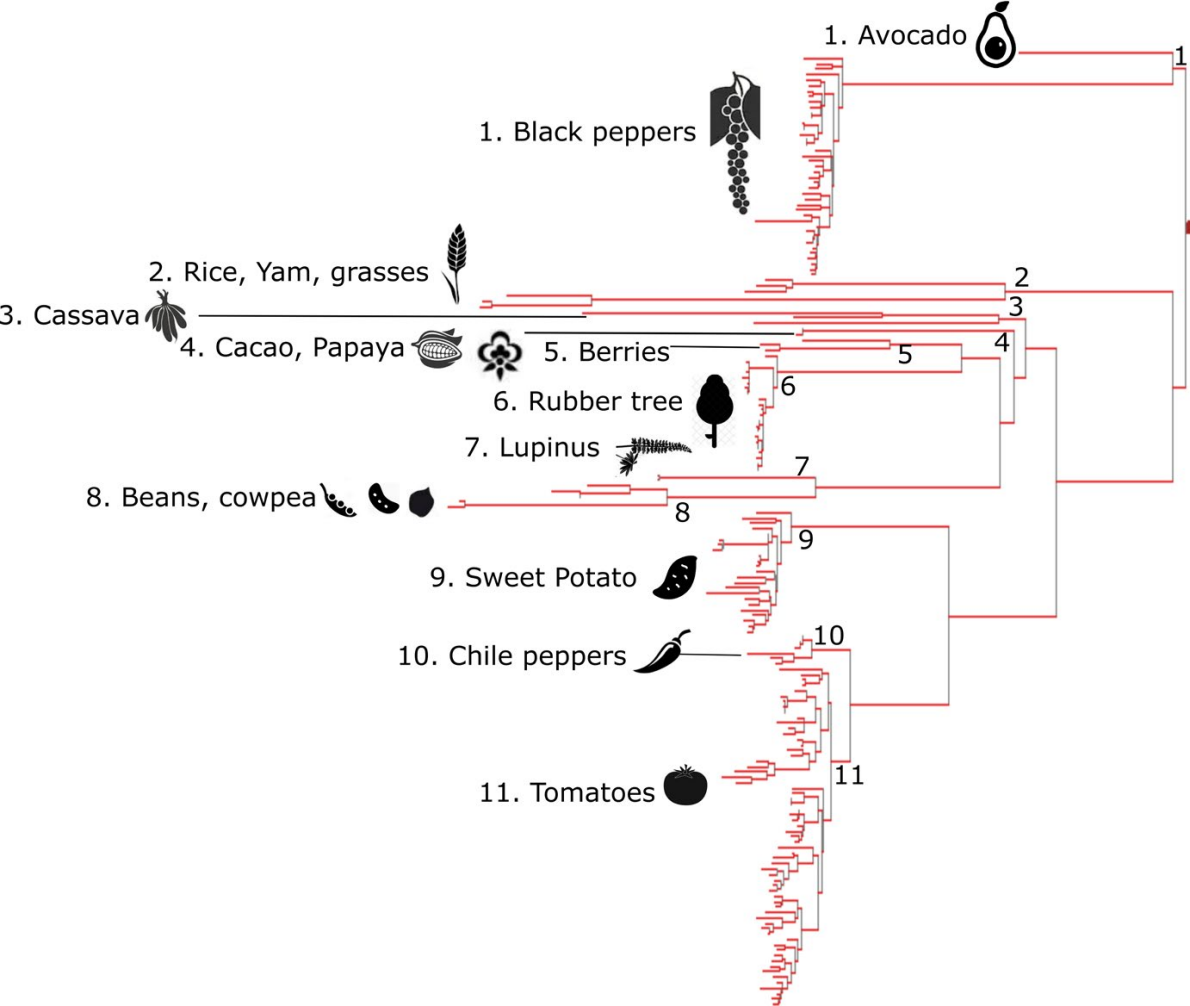
Phylogenetic tree of 185 species of CWR of Colombia representing 11 clades numbered as the main contributing groups

Our phylogenetic tree comprises 185 CWR species across 11 clades (Figure 1; Figure S2). The tree represents 17 of the 29 major gene pools of global crops prioritized by the CWR diversity project and recognized in the International Treaty on Plant Genetic Resources (Maxted & Kell, 2009).

### 3.2 Observed patterns of species diversity

**FIGURE 2:**
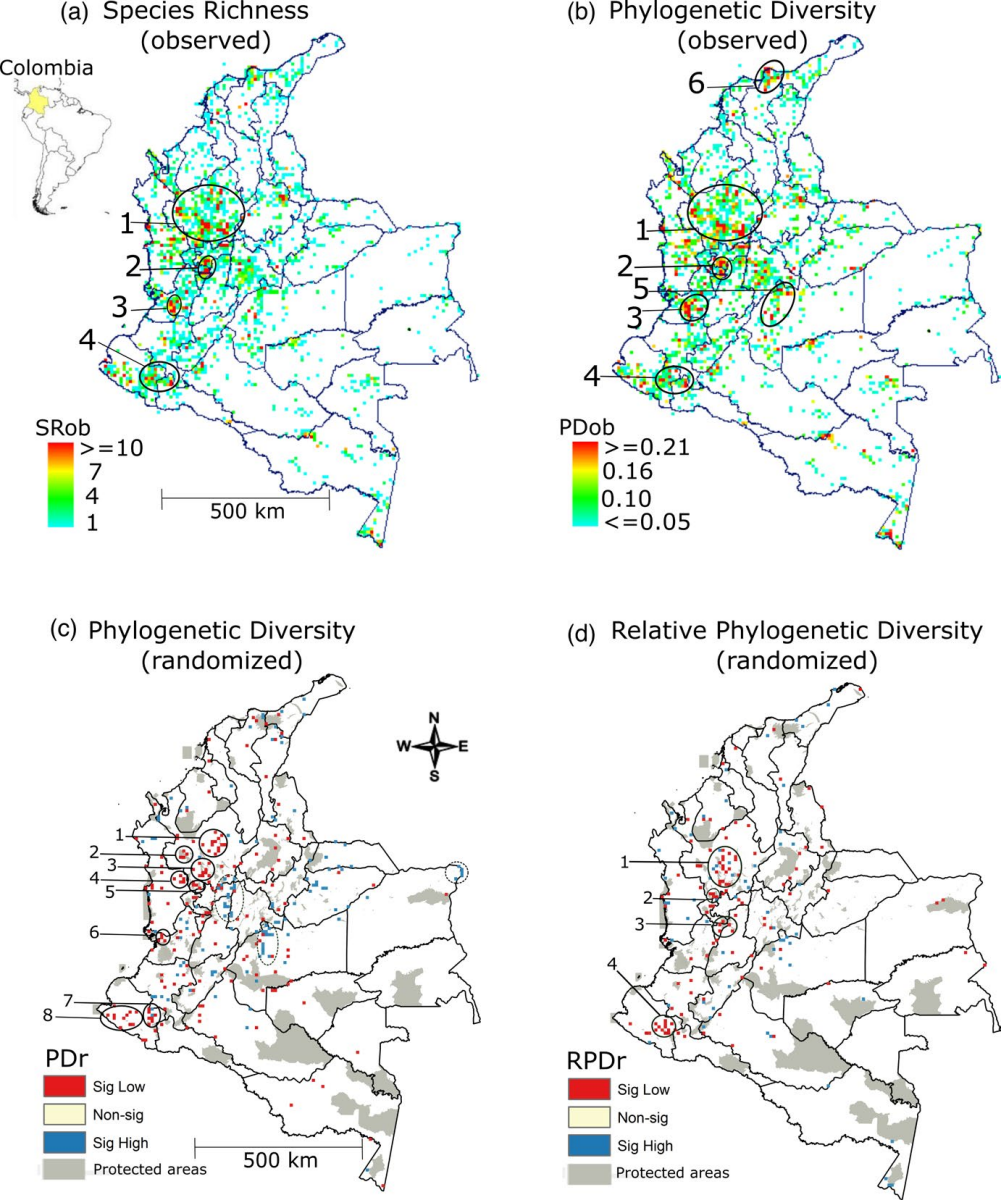
Spatial patterns of phylogenetic diversity of 185 species of crop wild relatives in Colombia. Map of South America showing the location of Colombia and observed species richness—SRob (a), observed phylogenetic diversity—PDob (b), randomised phylogenetic diversity—PDr (c) and randomized relative phylogenetic diversity (RPD)—RPDr (d). Locations with the highest concentration of diversity are numbered. Map 2a and 2b shows the 5–95 percentile of the highest values

We found four main regions of high concentration of CWR SR in Colombia, with the maximum value in a single cell being 31 species (Figure 2a). The largest is in the department of Antioquia, which is located on the northern part of the western and central mountain ranges (location 1 in Figure 2a). There are four sub‐groups within Antioquia, one in the northeast, two in the south and one on the western side of the department. The western slopes of the central ranges known as “Los Nevados” including the southern part of the Caldas department and borders between the departments of Quindío and Risaralda are the next highest area of high concentration of PD (location 2 in Figure 2a). The third region is in the inner slopes of the western range facing the Valle del Cauca region (location 2 and 3 in Figure 2a). The last region is part of the ‘Mazico Colombiano’ covering the slopes of the Galeras Volcano, part of Nariño and the region south of the town Mocoa in the Putumayo department (location 4 in Figure 2a). The redundancy analyses show that large parts of the Orinoquia and Amazon regions are poorly sampled, while the Andean region has high sample redundancy (Figure S1a).

### 3.3 Observed patterns of PD

We found that the mountainous regions in the northern and central mountain ranges of Colombia host the greatest amount of PD. Particularly, we identify four regions of high CWR PD in the Andean region of Colombia (locations 1–4 in Figure 2b). The region with the largest concentration of PD (41% of the tree) is near the tips of the eastern and central ranges (location 1 in Figure 2b). The areas of high PD values were very similar to those for SR (Figure 2b), with the exception of the main two areas (locations 5 and 6 in Figure 2b). The first is in the northern slopes of the ‘Sierra Nevada de Santa Marta’ in the Caribbean region, and the second is in the foothills of the Orinoquia region in the eastern range including the ‘Serranía de La Macarena’ (Figure 2b). There are also two smaller areas of high PD near the borders with Peru and Ecuador.

The overall pattern of PD in the Andean region of Colombia is one of significantly low values (Figure 2c). However, the main hot spot is in the Antioquia department, in the northern tip of the western and central Andean mountain ranges (locations 1, 2, 3 and 5 in Figure 2c). In the Choco department, the slopes facing the Pacific coast on the western range have a small area of significance (location 4 in Figure 2c). Locations 6 and 8 in Figure 2c are also part of the biogeographic Pacific region in the departments of Valle del Cauca and Nariño. The last group of significantly low PD is in the ‘Macizo Colombiano’ in Nariño department (location 7 in Figure 2c). These patterns of significantly low PD suggest that there is less of the tree than expected, and thus, most of the country contains taxa that are more closely related. Most of the significantly high PD values were scattered in the lowlands. However, one of the larger groups was found in the inter Andean valleys of the Magdalena River around Marquita, Honda in Tolima and ‘La Dorada’ in Caldas department (dotted ovals in Figure 2c). The second largest group of significantly high PD was found in the foothills of the Orinoquia region and La Macarena. One small group was in the ‘Puerto Carreño’ region of the Vichada department. These areas of high PD suggest that there is more of the tree and that those taxa were less closely related than would be expected by chance. We found incomplete representation of CWR PD in the Amazon and Orinoquia regions.

Four areas of significantly low RPD were found in places different to significantly low PD areas (locations 1–4 in Figure 2d). This suggests that CWR species in these three areas have shorter than expected branches, but are not necessarily more closely related than in the main centre of significantly low PD. These Andean sites might indicate places with the potential for evolutionary adaptation and speciation. The areas of significantly high RPD in the Andean region of Antioquia (location 1 in Figure 2d) indicate branches significantly longer than expected. There were also some scattered grid cells with significantly high RPD found in the eastern and northern parts of the country including the Orinoquia, Amazon and Caribbean regions (Figure 2d). These cases might indicate places with the potential for keeping unique evolutionary history because they have many long branches.

### 3.4 Conservation Gap analysis

**FIGURE 3:**
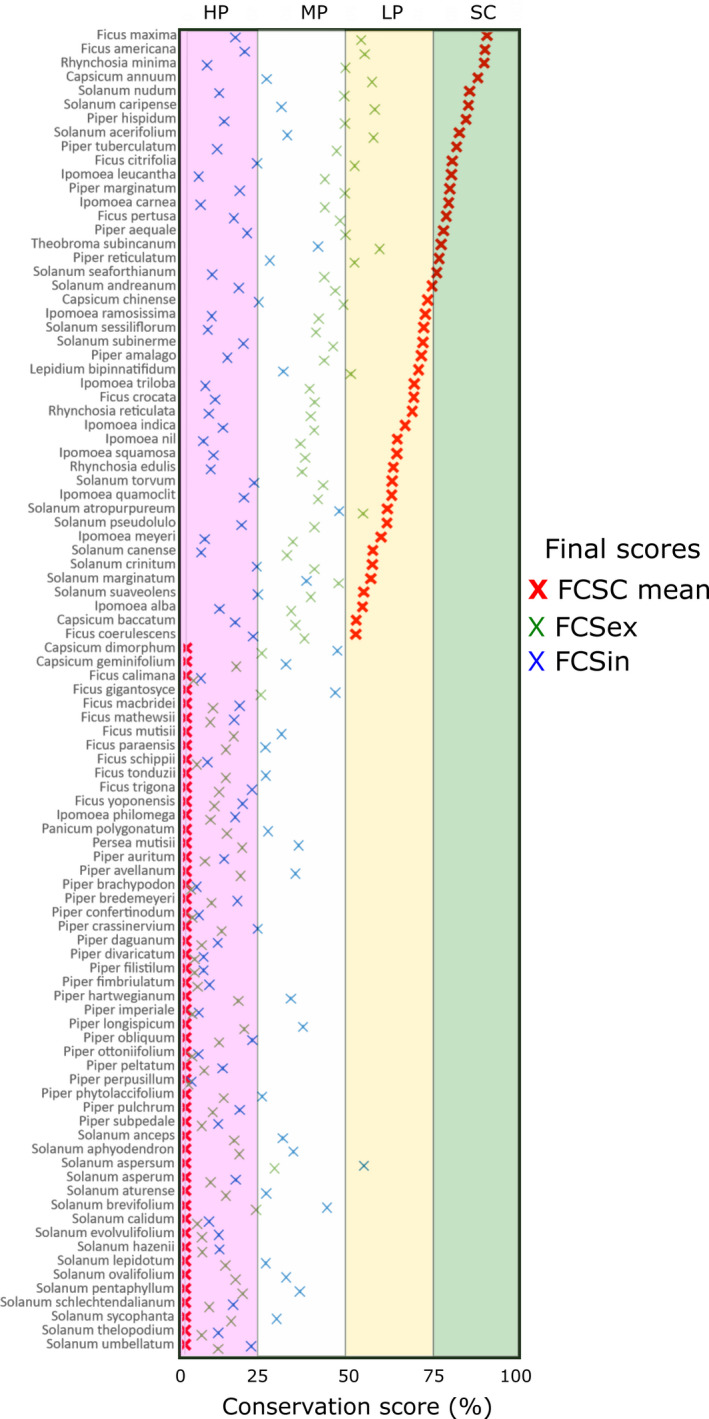
Conservation gap analysis results for 95 species of CWR in Colombia. Species are listed by ascending priority for further conservation action by priority categories (HP = high priority; MP = medium priority; LP = low priority; SC = sufficiently conserved). The red X represents the final conservation score combined (FCSc‐mean) for the species which is the average of the final ex‐situ (FCSex, green X) and in situ (FCSin, blue X) scores

The mean final conservation score (FCSc‐mean) across all species was 25.5 on a conservation status score of 0–25 (very poor) and 75–100 (comprehensive) with scores ranging from 0 to 90.96 (Table S3). The average ex‐situ conservation score across species was 33.05 and 18.05 for the in situ conservation (Figure 3). We found that 50 species (52.6%) were assessed as high priority, 36 (37.8%) medium priority and 9 (9.4%) low priority for further collecting to address gaps in ex‐situ conservation. For the case of in situ conservation, 73 (76.8%) were assessed as high priority, 21 (22.1%) medium priority and 1 (0.9%) low priority for further collecting to address gaps in in situ conservation.

The ecological representation of CWR species that have been collected for conservation repositories indicated to be a high priority, with a mean ecological representativeness score (ERSex) of 21.6, compared with 31.1 for the geographic score (GRSex). For the case of ex‐situ conservation, a total of 51 species (53.6%) were assessed as high priority, 26 (27.3%) low priority and 18 (18.9%) sufficiently conserved for further collecting to address gaps in ex‐situ conservation (Figure 3). The ecological representativeness (ERSin) of CWR species showed a low priority with a mean score of 76.9, compared with 17.4 for the geographic score (GRSin). For this case of in situ conservation, a total of 77 species (81%) were assessed as high priority, 23 (24.2%) medium priority and 1 (0.9%) low priority for further collecting to address gaps in in situ conservation (Figure 3). Although predicted ranges were high based on the ERSin, species were poorly represented in protected areas with the mean final in situ conservation score (FCSin) of 18.4. In contrast, FCSex presented an score of 33.1, suggesting that more effort need to be put on further collecting ex‐situ diversity of CWR outside protected areas.

### 3.5 Conservation assessment of PD

Twenty‐five of the 32 departments in Colombia had high PD grid cells inside protected areas. Despite showing a good spatial coverage of PD inside departments, more than half of the grid cells with the highest concentration of SR, PD and RPD of CWR were unprotected (55.9% of the total top scores), suggesting that only 44.1% of the highest concentration sites are inside protected areas (Table 1). The departments of Quindío, Risaralda and Córdoba showed the highest proportion of high PD representativeness with 70% of the largest concentration found inside protected areas. The departments on the eastern half of the country are the largest and least represented for conservation because they have a low percentage of PD inside and outside protected areas.

**TABLE 1:** CWR conservation assessment based on the percentage of observed species richness (SRob), observed PD (PDob), randomized PD (PDr), and randomized RPD (RPDr) hot spots per department present inside protected areas of Colombia

Departments	SRob (%) *N* = 118	PDob (%) *N* = 141	PDr Sig Low (%) *N* = 178	PDr Sig High (%) *N* = 108	RPDr Sig Low (%) *N* = 108	RPDr Sig High (%) *N* = 66	Averages per departments (%)
Quindío	100.0	100.0	66.7	100.0	66.7	100.0	88.9
Risaralda	0.0	80.0	100.0	100.0	100.0	100.0	80.0
Córdoba	100.0	100.0	100.0	100.0	66.7	0.0	77.8
Valle del Cauca	78.6	70.6	36.4	83.3	75.0	50.0	65.6
Boyacá	100.0	50.0	75.0	50.0	100.0	0.0	62.5
Magdalena	66.7	75.0	50.0	66.7	100.0	0.0	59.7
N. Santander	100.0	100.0	50.0	0.0	0.0	100.0	58.3
Amazonas	25.0	33.3	0.0	100.0	100.0	80.0	56.4
Cundinamarca	66.7	44.4	75.0	50.0	100.0	0.0	56.0
Caldas	50.0	50.0	66.7	42.9	50.0	50.0	51.6
Guajira	100.0	100.0	66.7	25.0	0.0	0.0	48.6
Meta	83.3	55.6	41.7	38.1	0.0	71.4	48.3
Antioquia	63.3	61.5	51.2	30.0	51.6	23.1	46.8
Santander	60.0	50.0	50.0	37.5	0.0	25.0	37.1
Sucre	100.0	100.0	0.0	0.0	0.0	0.0	33.3
Cesar	100.0	100.0	0.0	0.0	0.0	0.0	33.3
Putumayo	0.0	0.0	100.0	100.0	0.0	0.0	33.3
Guainía	0.0	0.0	0.0	0.0	100.0	100.0	33.3
Tolima	0.0	33.3	66.7	0.0	60.0	0.0	26.7
Huila	0.0	25.0	50.0	0.0	33.3	25.0	22.2
Choco	26.7	26.7	11.1	20.0	20.0	25.0	21.6
Nariño	25.0	55.6	14.3	0.0	26.7	0.0	20.3
Bolívar	0.0	0.0	0.0	100.0	0.0	0.0	16.7
Caquetá	0.0	0.0	30.0	0.0	20.0	25.0	12.5
Cauca	0.0	0.0	50.0	0.0	20.0	0.0	11.7
Total averages (%)	49.8	52.4	46.1	41.7	43.6	31.0	44.1

Abbreviation: CWR, crop wild relatives.

### 3.6 Phylogenetic diversity Gap conservation indicator

**FIGURE 4:**
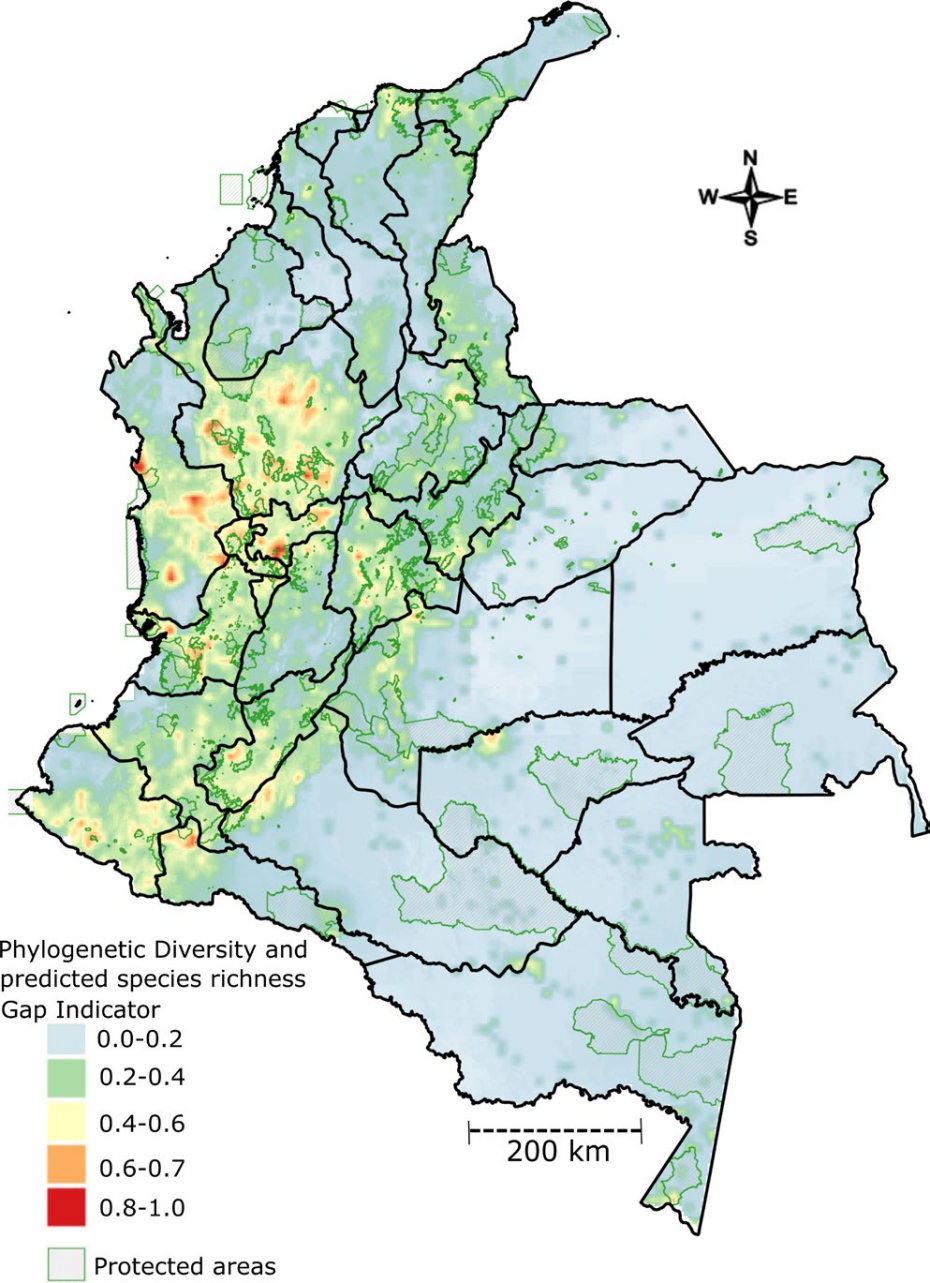
Map of phylogenetic diversity and predicted species richness gap indicator for identifying biodiversity hot spots of crop wild relatives in Colombia

The spatial distribution of PD and predicted species richness indicator shows that the mountainous areas of the Andean region have the highest concentration of PD and richness (Figure 4), Furthermore, the PD conservation gap richness indicators show similar regions as priority but these are poorly collected in the field (Figure S3). The main areas with high concentration of PD and richness are in the departments of Antioquia, Valle del Cauca, Choco, Caldas, Risaralda and Quindío in the northern and central regions of Colombia. The secondary high concentration areas are in the departments of Nariño, Caquetá, Huila and Cauca in the south of the country. Due to the low levels of ex‐situ collections, as well as high PD and species richness conservation gaps, these areas are suggested as the main candidates for further collecting of species CWR in Colombia.

## 4 DISCUSSION

Crop wild relatives are a priority because they are genetic resources for agriculture (Miller & Khoury, 2018). Despite recent advances in exploring native biodiversity (Aitken et al., 2008; Faith, 2013; Jarvis et al., 2008; Maxted, Avagyan, Frese, Iriondo, Kell, et al., 2015, Maxted, Avagyan, Frese, Iriondo, Magos, et al., 2015; Sgrò et al., 2011; Winter et al., 2013), an important but unanswered question is ‘What are the main consequences of PD loss?’ (Uchida et al., 2019). Most PD studies are focused on investigating PD directly associated with species of native flora, but more studies should be conducted linking CWR PD and conservation (Park et al., 2020; Zhang et al., 2015). According to the International Union for Conservation of Nature‐IUCN (2012), the branches of the tree of life are under imminent risk of extinction; thus, it is relevant to establish future conservation prioritization schemes taking PD into consideration (IPBES, 2019). A further research application of CWR PD is the generation of baseline information for plant breeding programmes. Particularly, exploring the PD of CWR is fundamental to identify cross‐compatibility between crops and their wild relatives which is an essential aspect for the future of agriculture (Viruel et al., 2021). Pironon et al. (2020) proposed the idea of unifying a concept of agro‐biodiversity when centres of diversity of wild and domesticated biodiversity are considered.

### 4.1 PD spatial distribution and centres

We analysed spatial patterns of PD for 185 CWR taxa and found novel areas of relevance to global CWR assessments (Figure 1). This sample allowed us to quantify for the first time the PD of CWR in Colombia. Despite this being the first attempt to build a phylogeny of CWR for Colombia using just the information from readily available and open access data sets, these 185 species of CWR can be considered as a sample of CWR prioritized under a global strategy and not as a nationally built species prioritization.

Our results demonstrate that there is one major area of CWR PD in the mountains of the northern part of the central and western ranges of Colombia (Figure 2a,b). There was a secondary area of high PD in the middle of the central range and the geographic region where the three Andean ranges join in the southwest of the country. This southern area of high PD diversity could be related to the fact that the western and central ranges converge in that area of high topographic and climatic heterogeneity.

The current spatial distribution results show that just a few speciose CWR groups such as figs, black peppers, sweet potatoes and tomatoes contribute the most to the formation of the main centres of PD and RPD (Figure 2c,d). Tomatoes were the main CWR found in the Andean region contributing to the areas of significantly low RPD (locations 1 and 2 in Figure 2d). These mid‐elevation areas (aprox. 1000–1500 masl) are semi‐rural landscapes in the north‐eastern and south‐western Andean regions of Antioquia characterized by small‐scale cattle ranching, plantain, cacao, coffee and sugarcane production. These regions with a high overall species diversity and CWR diversity can likely be conserved in some of the same places but special attention should be granted are areas with occurrence of particularly interesting CWR.

Black peppers were the main contributors to the areas with scattered grid cells of significantly low CWR RPD in the Pacific region of Choco (Figure 2d), which is part of an important global biodiversity hot spot (Myers et al., 2000). Cassava, sweet potato and fig or rubber trees were the main contributors to the areas with significantly high CWR RPD in the Pacific region of Choco (Figure 2d). Fig or rubber trees were the main contributors to the areas of significantly low CWR RPD in the Pacific region of Nariño, but also showed the presence of black peppers, sweet potato, chilli peppers and tomatoes (location 4 in Figure 2d). This area of Pacific lowlands could provide ideal conditions to further explore the cultivation and domestication of wild species of black pepper, which is a group of species that can be used by small‐scale agriculture. The Amazon region otherwise could provide a potential set of conditions and genetic diversity to establish future cropping areas, without deforestation, through domestication of these CWR. This is because they could be well adapted to extreme wet conditions in the lowlands that in some cases represent centres of diversity and origin such as for wild cacao (González‐Orozco, Sanchez, et al., 2020).

### 4.2 Centres of biodiversity and PD of CWR: Use in agriculture

Most of the literature on spatial phylogenetics focuses on the analysis of patterns of regional biodiversity (Baldwin et al., 2017; Dagallier et al., 2020; Garcia–R et al., 2019; González‐Orozco et al., 2015; Laffan et al., 2016; Mekala et al., 2019; Scherson et al., 2017; Sosa et al., 2018). There are comparatively few applications in the agricultural sciences (Jovovic et al., 2020; Martin et al., 2019; Perales & Golicher, 2014; Turley & Brudvig, 2016). PD can be an effective means to explore the effect of agriculture on evolutionary diversity; for example, Turley and Brudvig (2016) showed that landscapes with an agricultural history had a decrease in PD of plant communities. They found that plants became more closely related across time, leading to an increase in phylogenetic clustering, and suggesting a homogenization of the diversity of lineages in the tree of life.

Areas of high native biodiversity and agronomical resources of CWR could be a potential source of genetic resources well adapted and resilient to modern challenges (Pironon et al., 2020). In a recent study, González‐Orozco (2021) identified three main centres of species richness and 25 areas of high endemism for the native terrestrial species of plants found in Colombia.

In the same region of high diversity of the terrestrial flora of Colombia, we found areas of significantly high CWR PD with an over‐representation of short branches. Such patterns are indicative of phylogenetic clustering (Webb et al., 2002). Our results exemplified the ideas of Pironon et al. (2020) and Maxted and Vincent (2021) where different facets of native biodiversity and regions of importance for agriculture such as centres of crop origin or diversity converged making them of great importance for the future use of biodiversity (Pérez‐Escobar et al., 2019). Given that one of the centres of species richness for plants in Colombia found by Gonzalez‐Orozco (2021) was spatially congruent with the areas with the greatest CWR PD, policy and decision‐makers could consider using this information to establish centres of agro‐biodiversity in Colombia (location 1 in Figure 2). Specifically, these regions play a key role in the in situ conservation of native CWR PD of potential use for agriculture (Figure 4). Therefore, if these centres of CWR PD could be declared as important areas for maintenance of agro‐biodiversity, then it might lead to the development of conservation strategies of CWR in Colombia. Other ideas with similar vision have been developed in South America. For example, the institute of agricultural research of Peru (INAS) declared many areas in the country as zones of agro‐biodiversity.

### 4.3 Conservation of CWR species and PD

The taxonomic diversity of CWR included in this study represents 18 plant genera and 13 families (Table S2). *Solanum*, *Piper*, *Ficus* and *Ipomoea* are the genera that have the largest number of CWR species represented in the tree. There are still many other CWR taxa in Colombia that would require attention. It is therefore important that the taxa in Table 2 are used as a starting point in building further species lists for CWR prioritizations in Colombia.

**TABLE 2:** Preliminary list of some promising groups of useful plants for Colombia which could be considered as optional taxa of CWR (this list does not include exotic commercial flowers)

Family/common names	Genus	Type of crop
Passifloraceae (Passion fruit/Granadilla)	*Passiflora*	Fruit
Sapotaceae (Lucuma/Caimito)	*Pouteria*	Fruit
Faboidea (Guama)	*Inga*	Fruit
Annonaceae (custard apples/Guanabana or Cherimoya)	*Annona*	Fruit
Solanaceae (golden berries/tomatillo)	*Physalis*	Fruit
Myrtaceae (Guajaba/ Guayabo)	*Psidium*	Fruit
Caricaceae (carica/papaya)	*Carica*	Fruit
Cactaceae (prickly pear/tuna)	*Opuntia*	Fruit
Juglandaceae (walnut tree/nogal)	*Juglans*	Fruit
Amaranthaceae (velvet flower/amaranto)	*Amaranthus*	Cereal
Faboidea (red flower/bucare)	*Erythrina*	Legume
Faboidea (yellow sorrels/trebol)	*Oxalis*	Legume
Basellaceae (Ulluco)	*Ullucus*	Tuberous roots
Tropaeolaceae (nasturtium/mashua)	*Tropaeolum*	Tuberous roots
Apiaceae (Arracacha)	*Arracacia*	Tuberous roots
Asteraceae (ground apple/Yacon)	*Smallanthus*	Tuberous roots
Nyctaginaceae (umbellaworts/chago)	*Mirabilis*	Tuberous roots
Apiaceae (cilantro)	*Coriandrum*	Vegetables
Erythroxylaceae (coca)	*Erythroxylum*	Stimulants
Malpighiaceae (ayahuasca)	*Banisteriopsis*	Stimulants
Rubiaceae (Quinas/cascarillo)	*Cinchona*	Stimulants
Solanaceae (devils trumpet/campanillas)	*Datura*	Stimulants
Malvaceae (cacao de monte, cacaito)	*Theobroma, Herrania*	Stimulants
Anacardiaceae (Barniz, Cashew)	*Toxicodendron, Anacardium*	Resins and nuts
Arecaceae (palm trees)	*Astrocaryum, Bactris, Euterpe, Mauritia, Oenocarpus*	Fruit and oils

Abbreviation: CWR, crop wild relatives.

Overall, 52.6% of the wild relatives in this study were assessed as high priority for further preserving in situ and collecting for ex‐situ conservation. As additional metrics providing further detail to these results, we recommend using both the PD conservation gap richness and the PD‐predicted species richness.

The uncertainty of where to look for relevant regions of biodiversity is a disadvantage in hyperdiverse countries such as Colombia. Therefore, the first task is to identify areas of greatest diversity (González‐Orozco, 2021). However, to date, maps of critically important CWR PD have not been available for Colombia. The second task would be to establish the conservation status of species and regions of high PD that are protected. Prioritizing those regions by identifying areas with high concentrations of evolutionary relationships among CWR is an important contribution, particularly in the light of current deforestation and other human pressures on the natural ecosystems of the northern Andes (Figure 4).

The biogeographical regions that contain endangered dry forest were poorly represented by areas of high PD and gap richness indicator (Figure S3). However, some isolated pockets of dry forest had mid‐levels of PD (i.e. central inter Andean valleys in Antioquia, Santander and Norte de Santander, Arauca, Puerto Carreño–Vichada, Bolivar, Sucre, Cauca and Valle del Cauca). A specific CWR‐targeted collecting strategy for isolated semi‐arid and arid environments is highly recommended to improve ex‐situ conservation of genetic resources that may contain traits adapted to drought.

Informing conservation based on the results of the PD spatial patterns and species relationships is another use of the data (Table 1). For example, they could be used to create a discussion about which part of the tree of life is more strategic to preserve. However, it is not for us to decide which option is better or worse. Our PD results should be taken as a guideline but not a decision‐making tool. We could ask the question ‘would areas with long branches of limited evolutionary relationships be most important to conserve’? Or would areas with short branches of closely related evolutionary relationship and thus potentially a large amount of recent evolution/diversification be most important to conserve? We could argue that both are equally important because each of them represents a different facet of biodiversity and evolutionary history. In a country such as Colombia which has extremely high alpha diversity, a decision on protecting areas with a high concentration of closely related taxa would be a logical conclusion. This is the case of the Andean biogeographic region, which hosts most of the hyperdiverse genera of CWR in Colombia. For example, we found that the slopes of the western range showed the main areas of significantly low RPD, meaning a high concentration of short branches, that are closely related to the genera *Piper* and *Solanum*. If the aim would be to preserve a younger evolutionary diversification, then these places in those mountainous areas would be the best candidates. However, there are some cradles of important evolutionary diversity such as the Amazon and Orinoquia lowlands. In this case, we could say that preserving areas of significantly high RPD would be the best option for conservation of ancient biodiversity. Despite the low sampling in those regions, we identify a small number of sites with significantly high RPD in the eastern lowlands of the country where genera such as *Theobroma*, *Capsicum* and *Manihot* were found. In the context of a mega biodiverse country with many conservation priorities other than CWR, protecting biodiversity that is younger in origin is a better opportunity than concentrating on older evolutionary clades. However, some effort needs to be applied to strategic clades because losing ancient biodiversity such as the CWR genus *Theobroma* or cacao would imply compromising key biodiversity from nearer the root of the CWR tree of life.

### 4.4 Limitations: Undersampling and taxonomic biases

The genetic data used here are not considered as representative of all CWR present in Colombia. However, our sampling allowed us to build a phylogeny composed of 185 CWR taxa grouped in 11 major clades of global importance (Figure 1). The CWR species in the phylogeny were chosen based on an international database of CWRs (GBIF‐CIAT consortium), which is a valid species selection strategy. Consequently, further selection of CWR species used in PD analysis should consider using a more targeted sampling to improve the representation of native genetic diversity that is underrepresented in the international databases.

Colombia´s biogeographic regions are strongly influenced by elevation (González‐Orozco, 2021). A gradual increase in height above sea level generated drastic changes in vegetation zones in Colombia. We found that the main centres of PD and RPD (Figure 2c,d) follow specific elevational trends, which suggests that the CWR present there are adapted to different climate conditions. This study had better elevational representation of CWR from low to mid‐elevations (1000–1500 masl). Unfortunately, due to a lack of readily available data, the distribution of CWR in very high or low elevations is not as well sampled and are therefore underrepresented. In Colombia, the high‐altitude zones above 2000 masl are more impacted by land‐use changes due to the expansion of cities in the Andes. The other possibility is that less CWR diversity occurs in the highlands. The midlands (below 1500 masl) on the other hand have been more recently modified by human impacts such as deforestation (Etter et al., 2006, 2008). This indicates that the main centres of PD and RPD found at mid‐elevations are potentially under high risk of disappearance if population growth rates continue in the future. For the lowlands (below 1000 masl), we found areas of significantly high RPD, meaning that the CWR with long branches present there could be compromised by deforestation.

Our results suggest that Colombia´s CWR diversity is concentrated in a small number of wild crops such as legumes, tomatoes, black peppers and fig trees (Table S2). In the case of CWR, we found that approximately 23% of the country contains no spatial records at all. The Amazon, Orinoquia and Pacific regions of Colombia are undersampled, and therefore, its PD is poorly understood. These regions provide information about ancient species that are not necessarily domesticated yet but that were extensively used in agriculture by early human settlers (Lombardo et a., 2020). A new list of CWR for Colombia is required. CWR collections need a more intensive digitization process to enable straightforward access in a readily available format, something that could more generally lead to important scientific progress in countries where open information is often limited.

Taxonomically, we observed a sampling bias towards some of the major global food groups in Colombia (Table S2). For instance, the most common bias was to better sampled clades that belong to the Solanaceae (e.g. tomatoes). The remainder of the CWR clades require more data, by either an increased geographic coverage for the Amazon and Orinoquia regions or making more genetic sequences available in GenBank. It is important that more detailed analyses are done to better map the patterns at high elevations (above 2000 masl). Tackling this taxonomic bias requires new sampling campaigns and should be focused on unexplored groups of CWR, as suggested in Table 2. In contrast, the close relatives of the most traditional tropical crops in Colombia such as Cassava (*Manihot sculenta* L.) and common beans (*Phaseolus vulgaris* L.) are prime examples of well‐developed collections in Colombia (Chacon et al., 2008; Debouck et al., 1993; Ramirez‐Villegas et al., 2020). However, even for these well‐documented and comprehensive crop‐related groups, their data are not fully represented in the most common open‐source databases such as GenBank, GBIF and BIEN.

## 5 CONCLUSIONS

The CWR in the Amazon and Orinoquia regions are undersampled. The Andean mountains are the main reservoir of in situ and ex‐situ conservation of CWR PD in Colombia. Fifty‐two per cent of the CWR species ranked as ‘high priority’ and were poorly represented in germplasm databases and protected areas that possess the most ideal geo‐ecological conditions. The geographic gaps in both ex‐situ and in situ conservation of CWR largely aligned with areas of high concentrations of CWR PD and species richness. However, we found new areas of significant PD that are unexplored.

This study is the first attempt to quantify the PD of CWR in Colombia. We identified one major centre of PD on the northern part of the central and western ranges part of the Andean region of Colombia where we mostly found short branches. The other important areas of PD were in the Pacific region scattered in the southwest and the east of the country where we mostly found long branches in the lowlands. There is an urgent need to generate a list of CWR in Colombia that includes the non‐traditional and understudied wild crops. If not, we will continue to have an imbalanced understanding of CWR diversity in Colombia.

## CONFLICT OF INTEREST

The authors declare they have no conflict of interest regarding the data or inferences discussed in this manuscript.

## Supporting information

Figure S1

Figure S2

Figure S3

Table S2

Table S3

Dataset S1

Dataset S2

Supplementary Material

## Data Availability

The data that support the findings of this study are available in the [Link], [Link], [Link], [Link], [Link], [Link], [Link], [Link] of this article.
