# Supplementary figures and images for: Phylogenetic diversity and conservation of crop wild relatives in Colombia
Source: Evol Appl. 2021 Sep 16;14(11):2603–17. doi: 10.1111/eva.13295 (PMC8591330; doi:10.1111/eva.13295)

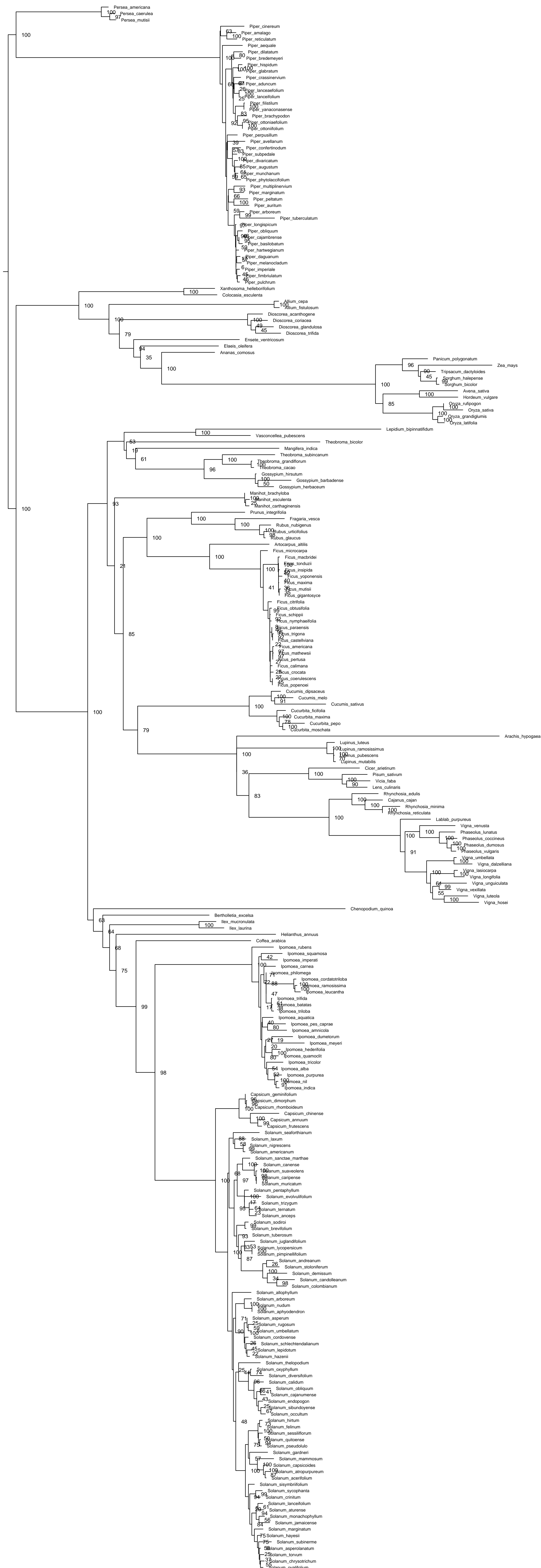

Supplement: Supplementary file 2 — Figure S2 [file EVA-14-2603-s007.pdf]

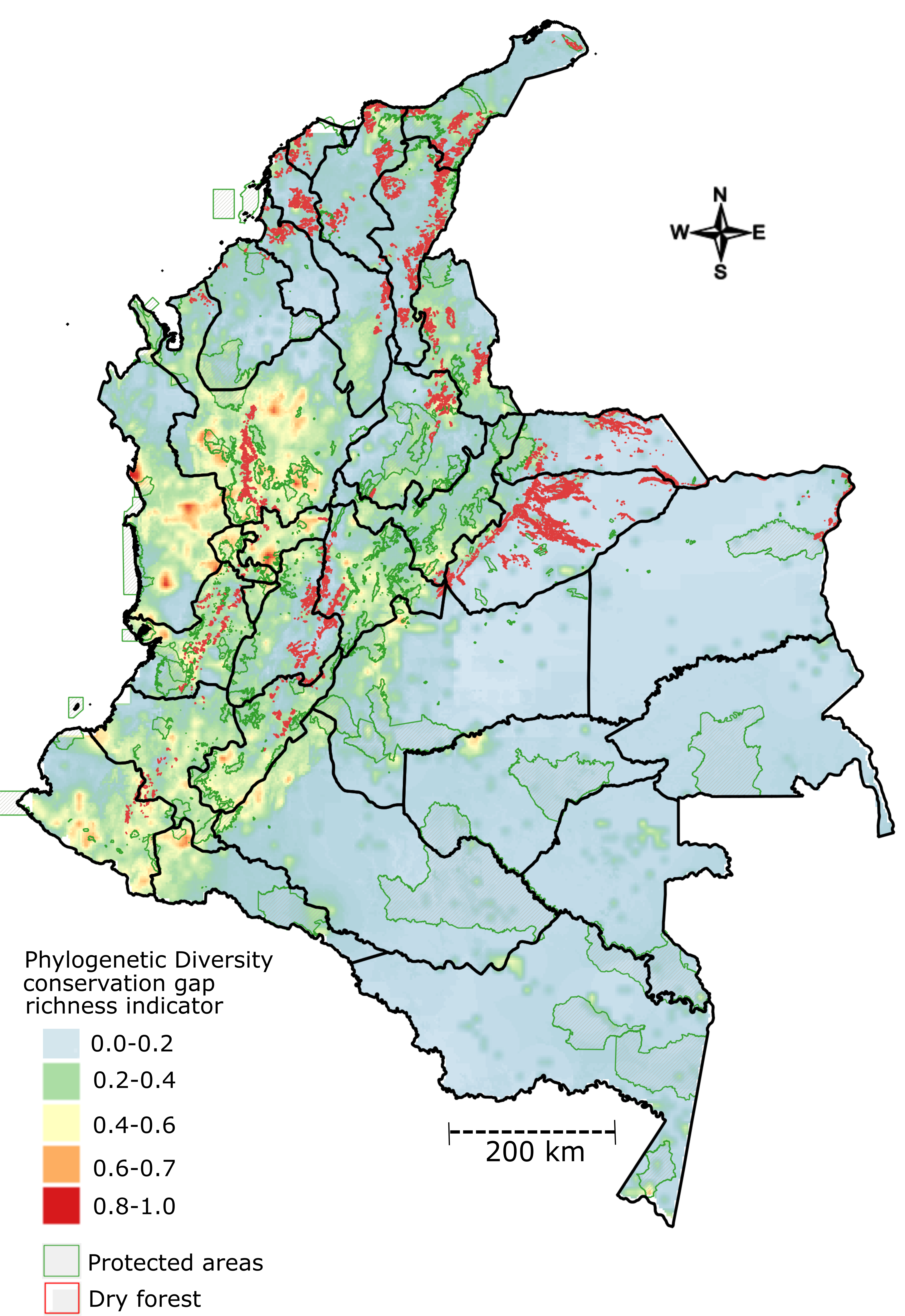

Supplement: Supplementary file 3 — Figure S3 [file EVA-14-2603-s003.png]
