# Supplementary material for: Phylogenetic diversity and conservation of crop wild relatives in Colombia
Source: Evol Appl. 2021 Sep 16;14(11):2603–17. doi: 10.1111/eva.13295 (PMC8591330; doi:10.1111/eva.13295)
Supplement: Supplementary file 8 — Supplementary Material [file EVA-14-2603-s001.docx]

**LIST OF SUPPLEMENTARY MATERIALS**

**Figure S1.** (a) Map of south America with the study region of Colombia and map of Colombia showing the values of sampling Redundancy (1 - [richness/#specimens]) where values close to 1 are well-sampled while zero means there is no redundancy in the sampling; (b) Digital Elevation Model of Colombia from Jarvis et al. (2008a) and (c) map and dendrogram showing the biogeographical provinces of Colombia according to González-Orozco (2021).

**Figure S2.** Colombia wild crops RAxML bipartition tree as a PDF (available as attached material).

**Figure S3.** Map of phylogenetic diversity conservation and gap richness indicator for further collecting ex-situ germplasm of crop wild relatives in Colombia.

**Table S1.** List of 185 CWR species used in our study.

**Table S2.** Species accessions for each locus used to develop the phylogeny (available as attached material).

**Table S3.** Results of the in-situ and ex-situ Gap Analysis for 95 species of CWR in Colombia (available as attached material).

**Dataset S1.** Species with family names and complete spatial dataset for species distributions (available as attached material).

**Dataset S2**. Phylogeny of Colombia´s CWR and some related crops (available as attached material).

**SUPPLEMENTARY MATERIALS**

**Figure S1.**

**
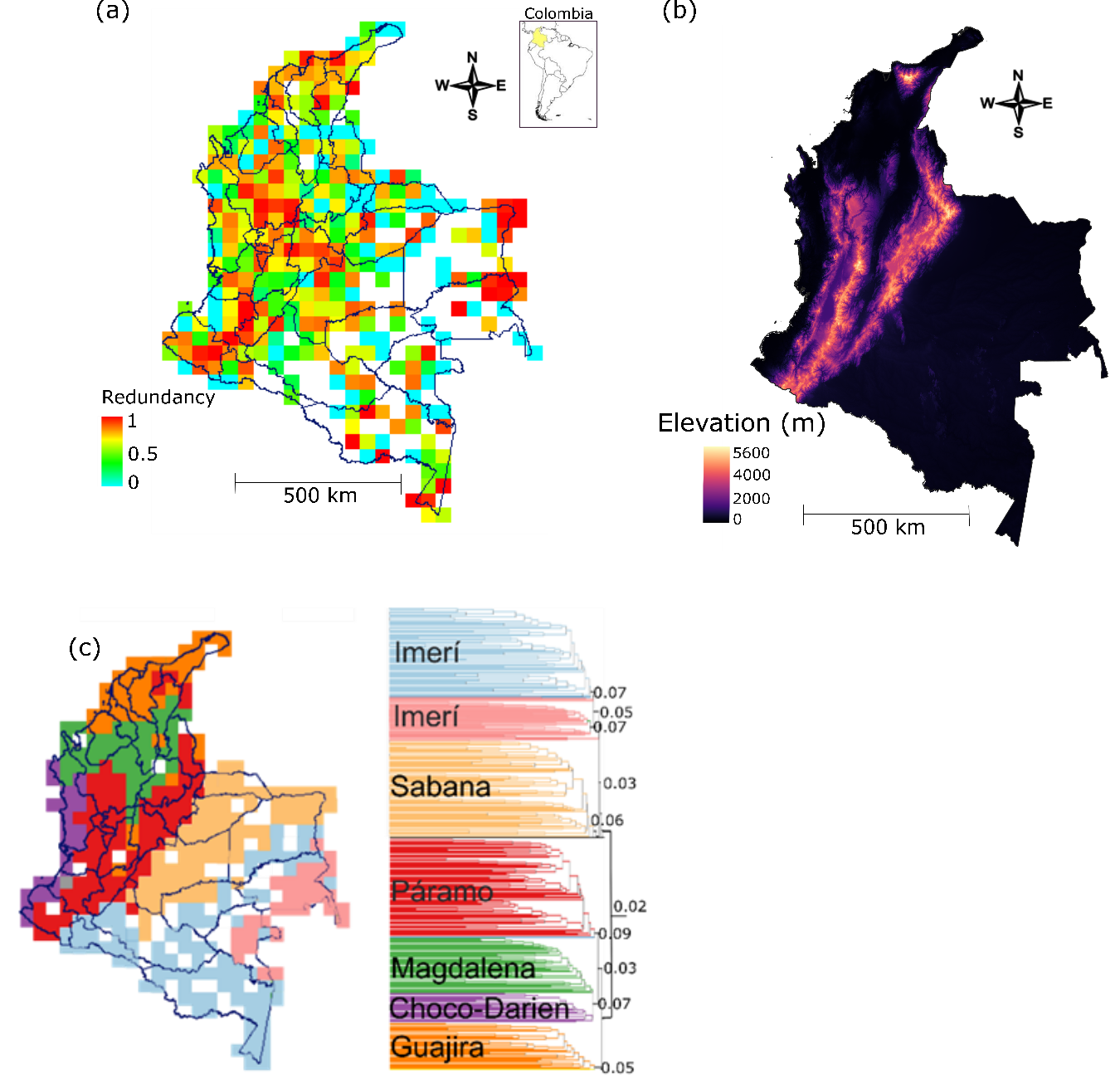
**

**Figure S3.**

**
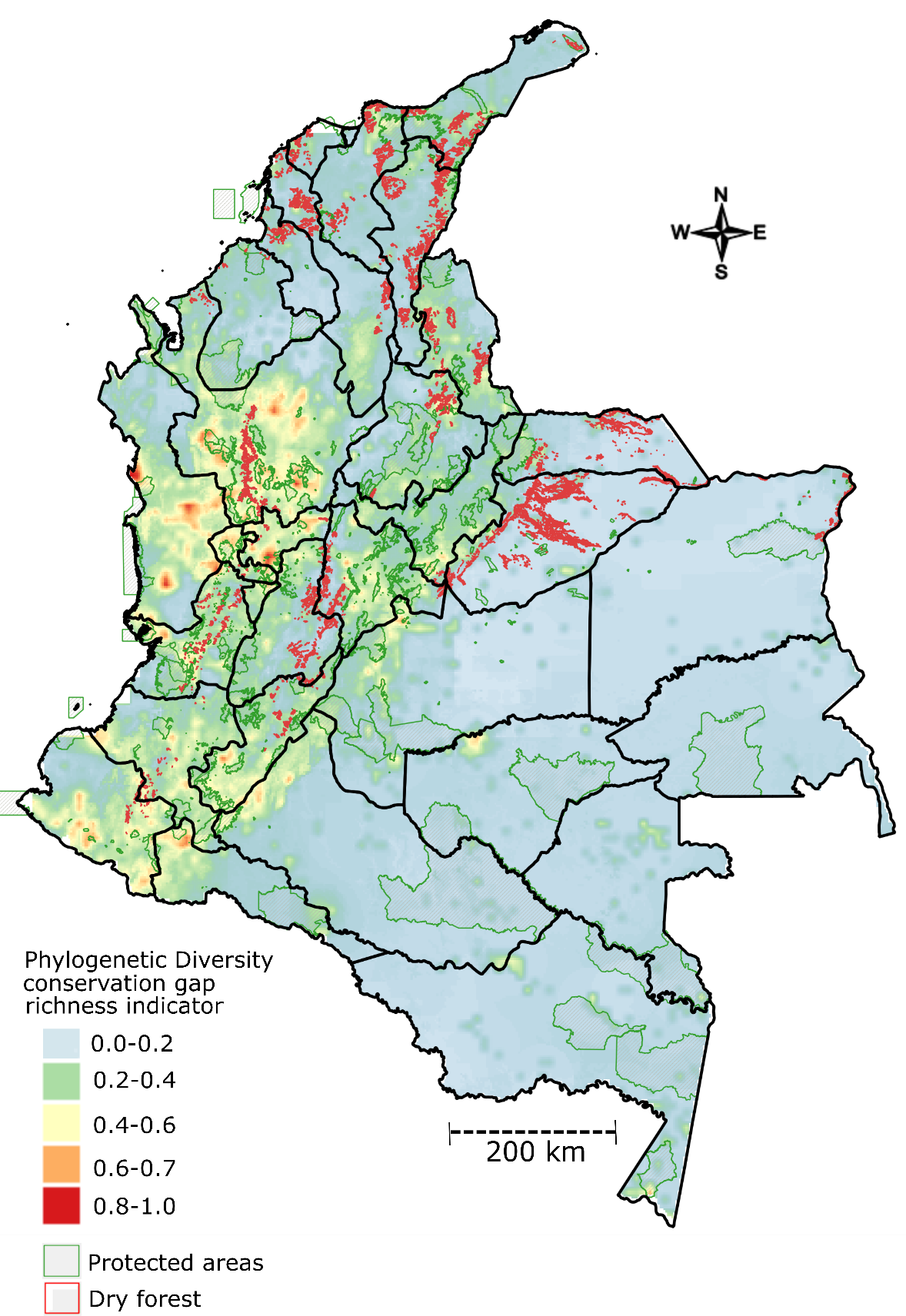
**

**Table S1.** List of 185 species used in our PD study with values of sampling redundancy.

| **Species name** | **VARIETY** | **SAMPLES** | **REDUNDANCY** |
| --- | --- | --- | --- |
| *Capsicum annuum* | 98 | 370 | 0.73513514 |
| *Capsicum baccatum* | 5 | 7 | 0.28571429 |
| *Capsicum chinense* | 33 | 177 | 0.81355932 |
| *Capsicum dimorphum* | 15 | 32 | 0.53125 |
| *Capsicum frutescens* | 1 | 1 | 0 |
| *Capsicum geminifolium* | 4 | 8 | 0.5 |
| *Capsicum rhomboideum* | 13 | 39 | 0.66666667 |
| *Dioscorea coriacea* | 1 | 1 | 0 |
| *Dioscorea glandulosa* | 1 | 1 | 0 |
| *Dioscorea trifida* | 1 | 1 | 0 |
| *Ficus americana* | 97 | 338 | 0.71301775 |
| *Ficus calimana* | 10 | 20 | 0.5 |
| *Ficus castellviana* | 2 | 2 | 0 |
| *Ficus citrifolia* | 46 | 105 | 0.56190476 |
| *Ficus coerulescens* | 8 | 10 | 0.2 |
| *Ficus crocata* | 16 | 34 | 0.52941176 |
| *Ficus gigantosyce* | 5 | 13 | 0.61538462 |
| *Ficus insipida* | 103 | 245 | 0.57959184 |
| *Ficus macbridei* | 19 | 33 | 0.42424242 |
| *Ficus mathewsii* | 18 | 33 | 0.45454545 |
| *Ficus maxima* | 83 | 199 | 0.58291457 |
| *Ficus microcarpa* | 3 | 4 | 0.25 |
| *Ficus mutisii* | 23 | 59 | 0.61016949 |
| *Ficus nymphaeifolia* | 48 | 82 | 0.41463415 |
| *Ficus obtusifolia* | 35 | 73 | 0.52054795 |
| *Ficus paraensis* | 41 | 82 | 0.5 |
| *Ficus pertusa* | 47 | 94 | 0.5 |
| *Ficus popenoei* | 2 | 2 | 0 |
| *Ficus schippii* | 21 | 51 | 0.58823529 |
| *Ficus tonduzii* | 39 | 92 | 0.57608696 |
| *Ficus trigona* | 30 | 68 | 0.55882353 |
| *Ficus yoponensis* | 13 | 23 | 0.43478261 |
| *Ipomoea alba* | 9 | 11 | 0.18181818 |
| *Ipomoea amnicola* | 3 | 4 | 0.25 |
| *Ipomoea aquatica* | 1 | 1 | 0 |
| *Ipomoea batatas* | 74 | 262 | 0.71755725 |
| *Ipomoea carnea* | 20 | 29 | 0.31034483 |
| *Ipomoea cordatotriloba* | 1 | 1 | 0 |
| *Ipomoea dumetorum* | 1 | 1 | 0 |
| *Ipomoea hederifolia* | 14 | 29 | 0.51724138 |
| *Ipomoea imperati* | 3 | 5 | 0.4 |
| *Ipomoea indica* | 18 | 29 | 0.37931034 |
| *Ipomoea leucantha* | 4 | 11 | 0.63636364 |
| *Ipomoea meyeri* | 4 | 6 | 0.33333333 |
| *Ipomoea nil* | 6 | 10 | 0.4 |
| *Ipomoea pes caprae* | 1 | 1 | 0 |
| *Ipomoea philomega* | 19 | 32 | 0.40625 |
| *Ipomoea purpurea* | 13 | 21 | 0.38095238 |
| *Ipomoea quamoclit* | 13 | 20 | 0.35 |
| *Ipomoea ramosissima* | 4 | 14 | 0.71428571 |
| *Ipomoea rubens* | 1 | 1 | 0 |
| *Ipomoea squamosa* | 15 | 23 | 0.34782609 |
| *Ipomoea tricolor* | 2 | 2 | 0 |
| *Ipomoea trifida* | 19 | 85 | 0.77647059 |
| *Ipomoea triloba* | 15 | 29 | 0.48275862 |
| *Lepidium bipinnatifidum* | 16 | 46 | 0.65217391 |
| *Lepidium bipinnatifolium* | 3 | 4 | 0.25 |
| *Lupinus pubescens* | 1 | 1 | 0 |
| *Lupinus puracensis* | 2 | 2 | 0 |
| *Lupinus ramosissimus* | 1 | 1 | 0 |
| *Manihot brachyloba* | 44 | 364 | 0.87912088 |
|  |  |  |  |
| *Manihot carthagenensis* | 18 | 146 | 0.87671233 |
| *Oryza grandiglumis* | 2 | 3 | 0.33333333 |
| *Oryza latifolia* | 38 | 402 | 0.90547264 |
| *Panicum polygonatum* | 15 | 33 | 0.54545455 |
| *Persea mutisii* | 28 | 79 | 0.64556962 |
| *Piper aduncum* | 101 | 303 | 0.66666667 |
| *Piper aequale* | 40 | 90 | 0.55555556 |
| *Piper amalago* | 21 | 36 | 0.41666667 |
| *Piper arboreum* | 75 | 214 | 0.64953271 |
| *Piper augustum* | 42 | 86 | 0.51162791 |
| *Piper auritum* | 25 | 53 | 0.52830189 |
| *Piper avellanum* | 6 | 11 | 0.45454545 |
| *Piper basilobatum* | 3 | 4 | 0.25 |
| *Piper brachypodon* | 16 | 56 | 0.71428571 |
| *Piper bredemeyeri* | 17 | 26 | 0.34615385 |
| *Piper cajambrense* | 2 | 3 | 0.33333333 |
| *Piper cinereum* | 18 | 44 | 0.59090909 |
| *Piper confertinodum* | 9 | 21 | 0.57142857 |
| *Piper crassinervium* | 48 | 178 | 0.73033708 |
| *Piper daguanum* | 7 | 17 | 0.58823529 |
| *Piper dilatatum* | 19 | 33 | 0.42424242 |
| *Piper divaricatum* | 8 | 11 | 0.27272727 |
| *Piper filistilum* | 9 | 25 | 0.64 |
| *Piper fimbriulatum* | 6 | 13 | 0.53846154 |
| *Piper glabratum* | 1 | 1 | 0 |
| *Piper hartwegianum* | 4 | 8 | 0.5 |
| *Piper hirsutum* | 1 | 1 | 0 |
| *Piper hispidum* | 49 | 134 | 0.63432836 |
| *Piper imperiale* | 19 | 43 | 0.55813953 |
| *Piper lanceaefolium* | 5 | 6 | 0.16666667 |
| *Piper lanceifolium* | 21 | 38 | 0.44736842 |
| *Piper longispicum* | 9 | 15 | 0.4 |
| *Piper marginatum* | 57 | 135 | 0.57777778 |
| *Piper melanocladum* | 10 | 15 | 0.33333333 |
| *Piper multiplinervium* | 27 | 47 | 0.42553191 |
| *Piper munchanum* | 19 | 68 | 0.72058824 |
| *Piper obliquum* | 64 | 147 | 0.56462585 |
| *Piper ottoniaefolium* | 3 | 3 | 0 |
| *Piper ottoniifolium* | 14 | 33 | 0.57575758 |
| *Piper peltatum* | 71 | 182 | 0.60989011 |
| *Piper perpusillum* | 4 | 6 | 0.33333333 |
| *Piper phytolaccifolium* | 15 | 23 | 0.34782609 |
| *Piper pulchrum* | 10 | 22 | 0.54545455 |
| *Piper reticulatum* | 26 | 77 | 0.66233766 |
| *Piper subpedale* | 10 | 20 | 0.5 |
| *Piper tuberculatum* | 47 | 83 | 0.43373494 |
| *Piper yanaconasense* | 1 | 2 | 0.5 |
| *Prunus integrifolia* | 23 | 61 | 0.62295082 |
| *Rhynchosia edulis* | 13 | 15 | 0.13333333 |
| *Rhynchosia minima* | 40 | 71 | 0.43661972 |
| *Rhynchosia reticulata* | 22 | 28 | 0.21428571 |
| *Rubus nubigenus* | 18 | 33 | 0.45454545 |
| *Rubus urticifolius* | 29 | 47 | 0.38297872 |
| *Solanum acerifolium* | 24 | 52 | 0.53846154 |
| *Solanum allophyllum* | 4 | 4 | 0 |
| *Solanum americanum* | 65 | 164 | 0.60365854 |
| *Solanum anceps* | 16 | 20 | 0.2 |
| *Solanum andreanum* | 16 | 126 | 0.87301587 |
| *Solanum aphyodendron* | 29 | 101 | 0.71287129 |
| *Solanum arboreum* | 42 | 139 | 0.69784173 |
| *Solanum asperolanatum* | 28 | 118 | 0.76271186 |
| *Solanum aspersum* | 4 | 4 | 0 |
| *Solanum asperum* | 10 | 19 | 0.47368421 |
| *Solanum atropurpureum* | 5 | 22 | 0.77272727 |
| *Solanum aturense* | 53 | 231 | 0.77056277 |
| *Solanum brevifolium* | 21 | 67 | 0.68656716 |
| *Solanum cajanumense* | 1 | 1 | 0 |
| *Solanum calidum* | 6 | 13 | 0.53846154 |
| *Solanum candolleanum* | 2 | 2 | 0 |
| *Solanum canense* | 4 | 8 | 0.5 |
| *Solanum capsicoides* | 3 | 4 | 0.25 |
| *Solanum caripense* | 23 | 82 | 0.7195122 |
| *Solanum colombianum* | 59 | 535 | 0.88971963 |
| *Solanum cordovense* | 7 | 16 | 0.5625 |
| *Solanum crinitum* | 16 | 42 | 0.61904762 |
| *Solanum demissum* | 1 | 2 | 0.5 |
| *Solanum diversifolium* | 7 | 9 | 0.22222222 |
| *Solanum endopogon* | 1 | 2 | 0.5 |
| *Solanum evolvulifolium* | 13 | 41 | 0.68292683 |
| *Solanum gardneri* | 5 | 9 | 0.44444444 |
| *Solanum hayesii* | 11 | 40 | 0.725 |
| *Solanum hazenii* | 12 | 26 | 0.53846154 |
| *Solanum hirtum* | 35 | 73 | 0.52054795 |
| *Solanum jamaicense* | 64 | 197 | 0.6751269 |
| *Solanum juglandifolium* | 34 | 180 | 0.81111111 |
| *Solanum lanceifolium* | 17 | 25 | 0.32 |
| *Solanum lanceolatum* | 1 | 1 | 0 |
| *Solanum lancifolium* | 1 | 1 | 0 |
| *Solanum laxum* | 4 | 5 | 0.2 |
| *Solanum lepidotum* | 33 | 106 | 0.68867925 |
| *Solanum mammosum* | 28 | 55 | 0.49090909 |
| *Solanum marginatum* | 5 | 9 | 0.44444444 |
| *Solanum monachophyllum* | 19 | 44 | 0.56818182 |
| *Solanum muricatum* | 2 | 3 | 0.33333333 |
| *Solanum nigrescens* | 24 | 69 | 0.65217391 |
| *Solanum nudum* | 41 | 138 | 0.70289855 |
| *Solanum obliquum* | 2 | 8 | 0.75 |
| *Solanum occultum* | 8 | 15 | 0.46666667 |
| *Solanum ovalifolium* | 35 | 79 | 0.55696203 |
| *Solanum oxyphyllum* | 2 | 13 | 0.84615385 |
| *Solanum pentaphyllum* | 12 | 26 | 0.53846154 |
| *Solanum pimpinellifolium* | 2 | 2 | 0 |
| *Solanum pseudolulo* | 13 | 22 | 0.40909091 |
| *Solanum rugosum* | 22 | 48 | 0.54166667 |
| *Solanum sanctae marthae* | 1 | 1 | 0 |
| *Solanum schlechtendalianum* | 17 | 28 | 0.39285714 |
| *Solanum seaforthianum* | 19 | 34 | 0.44117647 |
| *Solanum sessiliflorum* | 24 | 35 | 0.31428571 |
| *Solanum sibundoyense* | 3 | 5 | 0.4 |
| *Solanum sisymbriifolium* | 22 | 63 | 0.65079365 |
| *Solanum sodiroi* | 1 | 1 | 0 |
| *Solanum stoloniferum* | 1 | 1 | 0 |
| *Solanum suaveolens* | 5 | 6 | 0.16666667 |
| *Solanum subinerme* | 34 | 91 | 0.62637363 |
| *Solanum sycophanta* | 22 | 72 | 0.69444444 |
| *Solanum ternatum* | 4 | 7 | 0.42857143 |
| *Solanum thelopodium* | 15 | 32 | 0.53125 |
| *Solanum torvum* | 21 | 59 | 0.6440678 |
| *Solanum umbellatum* | 20 | 42 | 0.52380952 |
| *Theobroma subincanum* | 36 | 157 | 0.77070064 |
| *Vasconcellea pubescens* | 15 | 31 | 0.51612903 |
| *Vigna lasiocarpa* | 21 | 44 | 0.52272727 |
| *Vigna longifolia* | 5 | 5 | 0 |

**Table S2.** Taxonomic list of the studied genera of CWR for Colombia. The number of species per genus in indicated in brackets.

| Family/common names | Genus | Type of crop use |
| --- | --- | --- |
| Monocots |  |  |
| Poaceae  (rice, oats, grasses) | *Oryza* (2), *Panicum* (1) | Grains-cereals |
| Dicots |  |  |
| Brassicaceae  (peppergrasses) | *Lepidium* (2) | Food additives |
| Caricaceae  (papaya) | *Vasconcellea* (1) | Fruits |
| Convolvulaceae (sweet potato) | *Ipomoea* (23) | Tuberous |
| Dioscoreaceae (dioscorea) | *Dioscorea* (3) | Tuberous |
| Euphorbiaceae (cassava) | *Manihot* (2) | Tuberous |
| Fabaceae/Faboidea  (lupinus, cowpea) | *Lupinus* (3), Rhynchosia (3), *Vigna* (2) | Grains |
| Lauraceae (avocado) | *Persea* (1) | Fruits |
| Malvaceae  (cacao) | *Theobroma* (1) | Fruits |
| Moraceae  (Rubber trees) | *Ficus* (22) | Fruits |
| Piperaceae  (Black peppers) | *Piper* (42) | Food additives |
| Rosaceae  (stone fruits) | *Prunus* (1) | Fruits |
| Solanaceae  (chilli peppers, potatoes, tomatoes) | *Capsicum* (7), *Solanum* (66) | Fruits |
